# 12 Tips for Implementing Trauma-Informed Care Within Undergraduate Medical Education

**DOI:** 10.12688/mep.20612.1

**Published:** 2024-11-20

**Authors:** Donna Okoli, Margaret Dobson, Jill Schneiderhan, Molly Moravek, Jennifer Stojan, Mary Haas

**Affiliations:** 1 Emergency Medicine, University of Michigan Hospital, Ann Arbor, Michigan, USA; 2 Family Medicine, University of Michigan Hospital, Ann Arbor, Michigan, USA; 3 Obstetrics and Gynecology, Michigan State University College of Human Medicine, East Lansing, Michigan, USA; 4 Internal Medicine and Pediatrics, University of Michigan Hospital, Ann Arbor, Michigan, USA

**Keywords:** undergraduate medical education, trauma informed care, curriculum development

## Abstract

**Background:**

Trauma-informed care (TIC) has received increasing attention in the literature; however, implementation remains challenging and varies by the target learner group. Undergraduate medical education (UME) requires a longitudinal and broad-based approach to prepare students entering all specialties to incorporate TIC principles into daily practice.

**Aims and Methods:**

The Trauma-Informed Health Care Education and Research Committee (TIHCER) has released the first ever validated set of trauma-informed care competencies for undergraduate medical education, which serves as helpful framework for incorporation of TIC into UME curricula. A multi-specialty group of faculty clinical educators at the University of Michigan Medical School (UMMS) convened to articulate lessons learned from implementation of a longitudinal TIC curricula into the Doctoring clinical skills course.

**Results:**

Educators involved in designing and implementing TIC will find practical tips rooted in both literature and experience for successfully designing and actualizing trauma-informed care curricula in a longitudinal fashion.

**Conclusions:**

TIC can be successfully implemented and integrated longitudinally into UME. Efforts should include implementing the validated UME TIC competencies, strive to move on the continuum of TIC, and strategically partner with medical school leadership and institutional leadership to prioritize TIC efforts.

## Background

A growing body of literature recognizes the pervasiveness of trauma and its impact on patients. The Substance Abuse and Mental Health Administration (SAMHSA) defines trauma as “an event or circumstance that causes physical, emotional, or life-threatening harm, and has long lasting, negative effects on an individual's mental, physical, emotional, social or spiritual well-being ^1^. At least 70% of adults in the US have experienced at least one traumatic event in their lives ^2^. Trauma can generate subtle, insidious, or outright consequences for those who experience it ^3^. When unrecognized and unaddressed, trauma can lead to poor engagement in medical treatment and worse outcomes ^4^. An increased number of traumatic experiences correlates with increased rates of cancer, heart disease, substance use disorder, and psychiatric illness ^5^. Moreso, those who care for traumatized individuals, including medical students, can experience a phenomenon known as vicarious trauma ^6^. The impacts of vicarious trauma can impact student learning, well-being and performance ^6^.

TIC acknowledges the need to understand a patient’s life experiences in order to deliver effective care and has the potential to improve patient engagement, treatment adherence, health outcomes, and provider and staff wellness ^7^. Medical students equipped with knowledge and skills related to TIC may better recognize and address signs and symptoms of trauma amongst patients ^8^ and themselves.

The literature has offered descriptions of TIC in different settings: undergraduate medical education (UME), graduate medical education, and different medical specialties across the board ^9, 10^. However, challenges arise with actual operationalization of TIC due to lack of conceptual clarity and definitional understanding of TIC in the literature ^3^. Barriers to implementation include lack of: universal trauma precautions education; outcomes data; staff-focused interventions; and cost-effectiveness analysis ^9^. In 2022, the National Collaborative on Trauma-Informed Health Care Education and Research (TIHCER) released the first-ever validated set of TIC competencies for undergraduate medical education (UME) ^10^, augmenting the work of educators in this space. Other barriers include that changes made in the UME education have yet to be fully translated to the clinical environment ^9^. Additionally, educators should view TIC work on a continuum, rather than TIC education being regarded as binary; that curriculum is versus is not trauma-informed. Operationalizing TIC will require the strategic implementation of curricular change. Enhancing practical guidance for on the ground educators in UME may strengthen teaching of TIC principles and skills, leading ultimately to improved patient care for entire communities.

## Objectives

Herein, we provide 12 practical tips to facilitate longitudinal implementation of TIC curricula throughout the entirety of UME, to ensure that content is relevant to those entering all specialties and lay a solid foundation for further training in graduate medical education training programs.

### Tip 1. Create a multidisciplinary task force representing different specialties and experience levels

UME TIC curricular design requires a broad-based approach that emphasizes key principles relevant to all specialties. Accordingly, building a team of educators that includes stakeholders from a variety of specialties will enhance diversity of perspective and facilitate a more comprehensive approach. In addition to diversity among specialties, the team should include a combination of educators of different levels of seniority ^11^. Those closer to the experience of medical school may provide valuable perspective from the learner standpoint, as do those more senior with advanced clinical experience from the practitioner standpoint. This task force should also include students. Medical students offer perspective on current curricular issues and can give insight to how implemented changes might be perceived by the student body ^12^. Our workgroup at the University of Michigan includes a medical student, a medical education fellow, mid and senior career faculty. Specialties represented include emergency medicine, internal medicine, pediatrics, family medicine, and obstetrics and gynecology.

### Tip 2. Perform a needs assessment to identify areas of focus, current strengths and barriers to implementation at your institution

Specific institutions may vary in their current degree of integration of TIC principles into their medical school curriculum. While some have integrated TIC curricula for years and currently work to evaluate and refine them, others have just begun the design and implementation phase. Regardless of the institution’s particular spot on this continuum, performing a needs assessment can help educators and curriculum developers understand the current state of their learners’ knowledge and skills and provide direction for future initiatives. Needs assessments serve as the starting point for any curricular change ^13^. They can take the form of written surveys, interviews and/or focus groups, and should include a variety of stakeholders, including medical students of all class years, clerkship directors, medical school leadership (i.e. deans and course directors), faculty, and patients. Just as trauma reveals itself differently in each individual, individual institutions need to have a clear understanding of their specific needs in order to implement a successful curriculum. Specifically, at UMMS, students approached faculty with the desire to have TIC in their curriculum, which served as the catalyst for including TIC into the curriculum. It has now been integrated into the curriculum longitudinally and feedback from students and faculty have been used to modify the curriculum over time. Educators will need to gain access to time in the curriculum to implement TIC. Ensuring support from medical school leadership, including deans and course directors, will enhance success of TIC curriculum implementation.

### Tip 3. Join national communities of practice and utilize existing resources

Research involving teaching TIC in medical education represents a relatively new yet rapidly evolving field, ripe with opportunity for further growth and development. A robust national community of practice, entitled the National Collaborative on Trauma-Informed Health Care Education and Research (TIHCER), formed in 2018, includes a multidisciplinary group of health professionals “working to advance trauma-informed care across the continuum of practice through ground-breaking, transformative interprofessional education and research” ^14^. This collaborative has since expanded via word of mouth to include nearly 160 members located in 31 states and 5 countries representing 34 undergraduate medical education and residency programs, 7 pediatric clinics and hospitals, 7 psychiatry and behavioral health departments, 6 national councils and organizations, 5 Veteran Affairs (VA), 3 specialty clinics, 3 social change networks, 2 Departments of Health and Human Services, and 1 human information technology corporation ^14^. Interested educators can join the community through a link on the website ^15^, which also includes a repository of resources, including a TIC “toolkit” ^16^ that includes a list of TIC competencies, a bibliography with key literature, and a list of published TIC curricula. While one’s institutional workgroup can serve as a local community of practice, leveraging these connections and resources nationally can prevent educators from “re-inventing the wheel” and expedite knowledge translation from this content area.

### Tip 4. Utilize the CDC TIC framework to highlight trauma-informed principles throughout the curriculum

The CDC provides a framework for TIC that hinges on 6 core principles: safety; trustworthiness and transparency; peer support; collaboration and mutuality; empowerment, voice and choice; acknowledgement of cultural, historical and cultural issues ^17^. Educators can identify areas in their curriculum that already employ trauma-informed principles and make them explicit. For example, students are taught how to properly perform a physical exam, with emphasis on how to ensure that the patient remains autonomous and comfortable, a trauma-informed principle. Thus, effective physical exams are inherently trauma-informed. Many medical schools already teach such principles, but without explicit labeling within the TIC framework ^18^. Labeling such principles can facilitate movement towards true universal precautions.

### Tip 5. Integrate TIC into teaching of the history and physical exam in clinical skills courses

In addition to teaching medical knowledge around pervasiveness of trauma among patient populations, key concepts and definitions, and common triggers, TIC curricula must also emphasize the importance of incorporating principles into clinical skills such as taking histories and conducting physical exams. With regard to history taking, several resources exist for teaching students how to screen for trauma. In our experience, we found that teaching students to universally screen for trauma may not be feasible given time-constraints when they move into clinical practice. We have since emphasized that they should universally assume their patients have experienced trauma, be mindful of signs that indicate triggering or re-traumatization, and take the opportunity to dive deeper when pertinent and feasible. Regarding the physical exam, emphasizing both general and system-specific TIC principles is key. Several existing curricula and techniques published in the literature may provide guidance, for example: Soran ^19^, Elliseou *et al.* ^20^. In our clinical skills curriculum taught through the Doctoring course we have revised our teaching of the physical exam to include TIC language and skills beginning from the introduction and woven it throughout teaching each system-specific exam. Additionally, for institutions that incorporate peer physical exams, integrating TIC principles to avoid re-traumatization of students is also imperative as demonstrated in this curriculum by Elliseou *et al.* ^21^.

### Tip 6. Teach TIC in a longitudinal fashion

TIC should be foundational to training and educating future physicians, rather than a niche interest for opt-in by a cohort of physicians. Longitudinal learning leads to better development of essential skills. The Lancet Commission Report urges educational leaders to develop curricula that will serve patient and population needs, foster better understanding of the clinical context, emphasize continuous care over episodic encounters, and broaden training venues beyond inpatient care ^22^. Longitudinal learning allows for key concepts and principles to be repeatedly reinforced throughout one's education with the goals of foundational integration into one's practice of medicine. Teaching concepts earlier in the preclinical years allows students to fully integrate the principles into their practice of medicine. At our institution, TIC is taught longitudinally in the preclinical curriculum and threaded into early patient encounters and with standardized patients. Then, in the clinical years, sessions on TIC focus on more advanced and nuanced topics such as teaching self-regulation skills to patients and how to respond to a disclosure of trauma or to a trauma response during an encounter.

### Tip 7. Emphasize the aspects of TIC that are “physician facing”

We often focus on how TIC impacts patients, but it is meant to be protective for all who encounter patients, physicians included. Vicarious trauma or secondary traumatic stress is a phenomenon of caring for a traumatized person and can increase burnout among learners and practicing clinicians ^6^. Educating learners about the concept of vicarious trauma and strategies to address it may mitigate the consequences of caring for traumatized individuals. High rates of burnout, depression, and secondary traumatic stress that already plague medical students highlight the urgency of teaching coping strategies ^23^. Students foster resilience amongst themselves when school culture promotes peer support, mentorship, and extracurricular activities ^23^. Proven methods of coping with stress and psychological trauma include peer-to-peer based learning, reflection, collaborative small groups, and discussions for providers to debrief experiences at risk of causing vicarious trauma ^24^. Specifically, at UMMS, students attend a small group that meets once a week called “Safer Space” to facilitate these conversations and reflections.

### Tip 8. Use standardized patients to assess competency in TIC principles

UME is moving towards implementing competency-based education (CBME) through endorsement from the Association of American Medical Colleges (AAMC) ^25^. Competencies for TIC have been developed and validated by the TIHCER ^10^. Students should have multiple opportunities to demonstrate their competence through both simulated and real patient encounters. Standardized patient’s (SP) offer an excellent opportunity for students to simulate encounters prior to actually assessing real patients. Several curricula, such as those developed by Lee *et al.* and Lloyd *et al.* ^26, 27^ have used SPs specifically trained in TIC principles. This offers students a valuable opportunity to go beyond the learning about TIC to practicing the actual skills of TIC. This is a strategy to longitudinally build in universal precautions throughout a UME curriculum but also to create a TIC specific standardized patient assessment depending on the medical school’s curricular layout. In our program, we have SPs trained in TIC specifically for the sensitive exams. This has served as an excellent starting point in our curriculum. We are currently in the process of incorporating TIC into all standardized patient encounters. Next steps for the UME community at large include gathering consensus on entrustable professional activities (EPAs) that map to the TIC competencies.

### Tip 9. Aid students in accepting uncertainty and strive move along the continuum of TIC

Medical students often seek one correct answer. There is a growing body of literature in regard to how medical students respond to uncertainty. A trend that is being encountered with this current generation of learners is the request for an “answer key” for all aspects of their education ^28^. However, TIC requires elevating emotional IQ and using nonverbal cues to dive deeper into the nuances of the patient encounter, especially in an attempt to not retraumatize the patient in the encounter. This is difficult to teach– especially as these skills develop with time and increased interactions with patients ^28^. We specifically call out TIC curricular materials and name uncertainty and nuance as a strategy to help students acknowledge the potential tension and better cope with it. Longitudinal teaching of reflective skills and tolerance for uncertainty is supported in our institution through a 4-year small group cohort, with individual coaching. Ongoing work needs to advance tolerance for uncertainty to advance a truly trauma-informed culture in medical education. Moreso, the ethos of TIC is one of advancement. Rather than considering TIC curricula as binary (i.e. curriculum is or is not trauma-informed), consider where your curriculum is on the continuum of becoming more trauma-informed.

### Tip 10. Provide faculty development opportunities for clinical educators engaged in UME teaching

Equipping faculty to build their working knowledge and commitment to teaching trauma-informed care requires that there are opportunities for them to develop professionally in this area. Students can be taught TIC principles in the preclinical years, but they need to see TIC practiced once they move into clinical training for it to actually be reinforced. Many educators who will be called upon to teach TIC curricula may not have had any formal training in TIC themselves ^29^. Adequate dissemination of TIC knowledge to students will require that time and formal education/training is given to faculty to ensure they are prepared to do so. In addition, faculty development efforts can empower educators to approach their educational work broadly with a trauma-informed lens. There is a framework within educational spheres called trauma-informed pedagogy which advances the understanding that applying trauma-informed principles in education acknowledges and mitigates the impact of trauma on learning ^30^. Faculty with foundational understanding of the components of TIC will be able to highlight, re-emphasize, and design TIC curricula for all areas of instructional content including clinical education. The goal is to have curricular alignment with other content, including but not limited to social determinants of health, anti-racism, and structural competency ^10^. We have developed a faculty development session that combines TIC with trauma-informed pedagogy for all faculty who teach clinical skills. This session is an introduction to considering their own clinical practice, their clinical teaching and finally their educational work through a trauma-informed lens.

### Tip 11. Strategically partner with medical education leadership and institutional leadership to prioritize TIC efforts

Systemic and institutional change is required for TIC to truly have impact in educational and clinical environments. Trauma-Informed Medical Education (TIME) establishes that students and trainees can experience trauma from a biased medical system ^31^. TIME promotes the creation of policies and practices that support learners to prevent re-traumatization as a means to deliver equitable education. This approach requires institutions to understand learners’ experience with bias and discrimination in medical education. TIME urges institutions to recognize and acknowledge the implicit racial bias in the student- instructor relationship, bias in the learning environment, and bias that students may experience from patients and their families ^31^. TIME proposes institutional and organizational change that leads to a more inclusive and diverse learning environment and medical education system. When you make changes to medical education, changes to the clinical environment should follow suit, so as not to undermine the work being done in UME. Tufts University provides an inspiring example through explicitly calling out a trauma-informed practice in both medical education and in clinical practice ^32^. They have crafted three pillars for a trauma-informed practice that encompass change both in medical education and patient care. These pillars are healing centered engagement, culturally sustaining pedagogy, and universal design for learning.

### Tip 12. Drive TIC work forward in UME with the creation of validated assessments

A lack of outcomes data often challenges the implementation of TIC curricula. Literature surrounding TIC mainly focuses on describing TIC as a framework. One systematic review found a lack of robust empirical research literature in TIC in relation to the number of theoretical commentaries that have been published ^33^. Defining and collecting relevant outcomes data will advance the understanding of optimal TIC curricula. Previously published TIC assessments have largely included knowledge-based exams. Educators should turn their attention to developing rigorous assessment criteria and rubrics, specifically objective, skill-based assessments that map to the recently established TIC competencies ^29^.

## Conclusion

The 12 tips outlined above serve as a guide for medical educators to longitudinally implement TIC into their UME curriculum. As the literature has established that trauma has significant health effects, medical educators must prepare medical students to employ TIC principles. Educators can now focus efforts to integrate TIC principles with the use of the newly validated TIC competencies for UME. Collaboration and learning from existing efforts can advance these principles and lead to the development assessments to understand their impact. As TIC curricula are formally established and expanded within medical schools, educators should engage in program evaluation, both on individual, institutional, and national levels.

## Ethics and consent

Ethical approval and consent were not required.

## Data Availability

No data are associated with this article.
