# Supplementary material for: 12 Tips for Implementing Trauma-Informed Care Within Undergraduate Medical Education
Source: MedEdPublish (2016). 2024 Nov 20;14:281. [Version 1] doi: 10.12688/mep.20612.1 (PMC12572772; doi:10.12688/mep.20612.1)
Supplement: Peer review history

# Reviewer report: Reviewer response for version 1

**DOI:** 10.21956/mep.22065.r42316

**Published:** 2025-11-05

**Authors:** Saima Batool

**Affiliations:** 1 University of Lahore, Punjab, Pakistan

**Constructive Feedback on “12 Tips for Implementing Trauma-Informed Care Within Undergraduate Medical Education”**

**What was done well**

 The manuscript makes a highly valuable and timely contribution to the field of undergraduate medical education (UME) by presenting a structured, evidence-informed framework for implementing trauma-informed care (TIC). The document is rooted in both literature and lived faculty experiences. The inclusion of validated TIC competencies from the Trauma-Informed Health Care Education and Research Committee (TIHCER) lends credibility and alignment with national best practices.

 The authors also effectively contextualize the urgency and relevance of TIC in UME, providing a strong theoretical background grounded in SAMHSA and CDC frameworks. The paper’s longitudinal approach—threading TIC throughout the curriculum and emphasizing faculty development, standardized assessments, and institutional alignment—reflects best practices in curriculum integration and change management.

 Importantly, the emphasis on **both patient-facing and physician-facing aspects of TIC** (e.g., addressing vicarious trauma in students) demonstrates an inclusive and holistic view of medical education. The inclusion of peer-reviewed literature and national collaborations (e.g., TIHCER, TIME) shows scholarly depth and advocacy for systemic change.

**How to improve**

 To enhance impact, the authors may consider:

1. Adding **brief institutional case studies** from different geographic or resource settings to increase applicability.
2. **Incorporating visual summaries** (e.g., infographic of the 12 tips, TIC implementation roadmap, competency alignment matrix).
3. Providing **barrier-solution pairs** in a tabulated format to guide implementers through common challenges.
4. Expanding the **assessment strategy** section with specific examples of OSCE station rubrics or reflective portfolios aligned with TIC.
5. Embedding **learner and patient voices** directly through quotations or brief reflections.

If evidence from practice is presented, are all the underlying source data available to ensure full reproducibility?

Yes

Is the topic of the practical tips discussed accurately in the context of the current literature

Yes

Are all factual statements correct and adequately supported by citations?

Yes

Are the conclusions drawn balanced and justified on the basis of the presented arguments?

Yes

Are arguments sufficiently supported by evidence from the published literature and/or the authors’ practice?

Yes

Reviewer Expertise:

Pediatrics, Professionalism, simulation, gamification, curriculum, assessment, EDI, professional and personal identity formation,

I confirm that I have read this submission and believe that I have an appropriate level of expertise to confirm that it is of an acceptable scientific standard.

# Reviewer report: Reviewer response for version 1

**DOI:** 10.21956/mep.22065.r43710

**Published:** 2025-10-29

**Authors:** Kathryn Young

**Affiliations:** 1 Metropolitan State University of Denver, Denver, Colorado, USA

This is very well written and makes a lot of sense. There are a few instances where some editing would make a few sentences clearer but other than that the article is impressively written and meets the goals it set out for itself. I also appreciate the step-by-step approach to TIC implementation from the individual level, to coursework, to policies, to making sure senior leadership is involved.   My only query is that in TIP 11, in the section on bias, I wonder if you want to include bias from other medical students in the program. This is not necessary, but I know in other fields that other students are sometimes the ones who present the most bias.   I think practitioners will find this article very useful and actionable!

If evidence from practice is presented, are all the underlying source data available to ensure full reproducibility?

Yes

Is the topic of the practical tips discussed accurately in the context of the current literature

Yes

Are all factual statements correct and adequately supported by citations?

Yes

Are the conclusions drawn balanced and justified on the basis of the presented arguments?

Yes

Are arguments sufficiently supported by evidence from the published literature and/or the authors’ practice?

Yes

Reviewer Expertise:

trauma-informed practices in higher education, hierarchical microaggressions

I confirm that I have read this submission and believe that I have an appropriate level of expertise to confirm that it is of an acceptable scientific standard.

# Reviewer report: Reviewer response for version 1

**DOI:** 10.21956/mep.22065.r40200

**Published:** 2024-12-16

**Authors:** Shahrzad Bazargan-Hejazi

**Affiliations:** 1 Charles R. Drew University of Medicine and Science and UCLA David Geffen School of Medicine, Los Angeles, USA

The article *'12 Tips for Implementing Trauma-Informed Care Within Undergraduate Medical Education' *delves into this crucial topic, advocating for the systematic integration of TIC principles into medical curricula. It begins by defining TIC and its impact, citing evidence of trauma's pervasive effects on patient engagement and recovery. The Trauma-Informed Health Care Education Research Committee (TIHCER) provides a structured outline of TIC competencies, drawing on lessons from their application at the University of Michigan Medical School to illustrate successful long-term integration across training programs.

 Key strategies discussed include establishing a multidisciplinary task force, engaging in thorough needs assessments, and leveraging the CDC's TIC framework. These steps are underscored by the importance of using standardized patients in clinical training to ensure that future medical professionals can competently apply TIC approaches. The article also emphasizes the necessity for ongoing faculty development and strategic leadership collaboration to overcome potential implementation challenges and sustain TIC initiatives.

 While the article aligns well with contemporary literature and provides reproducible evidence and well-justified conclusions, there is room for enhancement. The authors could improve the piece by incorporating more detailed case studies or examples from various medical schools to illustrate the implementation process more vividly, expanding discussions on potential barriers and challenges with practical solutions, and including a broader range of stakeholder perspectives to enrich the article’s depth. Additionally, updating and broadening references to include the latest research and adding visual aids such as charts or graphs could strengthen the article’s authority and aid reader comprehension.

 Overall, this is a commendable contribution to medical education literature, promoting a paradigm shift towards more empathetic and effective patient care. It serves as a call to action for medical educators to champion the integration of trauma-informed practices into their teaching and clinical environments, ensuring a holistic approach to health care education.

If evidence from practice is presented, are all the underlying source data available to ensure full reproducibility?

Yes

Is the topic of the practical tips discussed accurately in the context of the current literature

Yes

Are all factual statements correct and adequately supported by citations?

Yes

Are the conclusions drawn balanced and justified on the basis of the presented arguments?

Yes

Are arguments sufficiently supported by evidence from the published literature and/or the authors’ practice?

Yes

Reviewer Expertise:

Mental Health and Research Education

I confirm that I have read this submission and believe that I have an appropriate level of expertise to confirm that it is of an acceptable scientific standard.
